# Evaluation of the removal ability of Iodoform Paste in primary teeth pulpectomies using rotary files: a pilot study

**DOI:** 10.1038/s41598-026-42179-7

**Published:** 2026-03-02

**Authors:** María Fe Riolobos González, Anabella Reyes Ortiz, María Lourdes García-Navas Fernández de la Puebla, Marta Macarena Paz-Cortés, Andrea Martín-Vacas

**Affiliations:** 1 Facultad de Odontología, Universidad Alfonso X El Sabio, Villanueva de la Cañada, 28691 España; 2 Máster en Odontopediatría, Centro Odontológico de Innovación y Especialidades Avanzadas, Universidad Alfonso X El Sabio, Madrid, 28037 España; 3 Departamento de Especialidades Clínicas Odontológicas, Facultad de Odontología, Universidad Complutense de Madrid, Madrid, 28040 España

**Keywords:** Pediatric dentistry, Pulpectomy, Dental care, Primary tooth, Diseases, Health care, Medical research

## Abstract

The aim was to analyse the efficacy of Endogal Kids^®^ rotary files in removing root canal filling. Methods: A cross-sectional in vitro study was conducted (*n* = 10 primary teeth). The root canals were instrumented using the Endogal Kids^®^ system with 5 mL of 5% sodium hypochlorite solution and were obturated with CaOH2 and iodoform paste, Forendo™ Paste. The canal systems were retreated to remove the filling material using continuous rotation. µCT scans were performed at three time points: after canal instrumentation with rotary files (µCT-1), after root canal filling (µCT-2), and after removal of the filling material through retreatment (µCT-3). Canal void volume (CV), obturation material volume (MV), root canal void increase (ΔCV), obturation material increase (ΔMV) and filling removal volume (RV) were calculated. Statistical analysis was performed with a 95% significance level (*p* ≤ 0.05). Results: Significant differences were found for CV and MV, but not for TV. The canal void volume (CV) was significantly greater at µCT-1 compared to the µCT-2 and µCT-3, with no significant difference between the µCT-2 and µCT-3 (*p* < 0.001). Additionally, MV was significantly higher before removal of the filling material (*p* = 0.003). No significant differences were observed in TV across the three measurements. Conclusions: This pilot study demonstrated that rotary instrumentation in primary teeth can remove a significant proportion of iodoform-based obturation material. Incisors showing greater obturation material removal, while molars exhibited more pronounced changes in canal volume.

## Introduction

Dental caries and traumatic injuries in pediatric patients may compromise the integrity of the pulp tissue, potentially leading to pulp necrosis or irreversible pulpitis. Pulpectomy, a root canal procedure performed on primary teeth, plays a crucial role in preserving deciduous dentition. This intervention helps prevent the development of deleterious oral habits, maintains arch integrity by avoiding premature space loss, and supports the preservation of essential oral functions^1^. The primary objective of pulpectomy is to achieve thorough disinfection of the root canal system and to establish a hermetic seal that prevents microbial reinvasion^2^.

Several factors influence the success rate of pulp therapy in primary teeth, among which the morphological complexity of the root canal system, both internal and external, is particularly significant^3^. Such anatomical intricacies increase the unpredictability and technical difficulty of the procedure^1^. Determining the apical endpoint, or working length (WL), is often challenging due to the frequent absence of a defined apical constriction^4^. Histological studies have identified that the primary causes of pulpectomy failure are technical limitations arising from irregularities induced by internal or external root resorptions, which are often associated with residual necrotic or bacterial remnants within the canal system following treatment^5^. Consequently, thorough mechanical and chemical preparation is essential, along with the use of filling materials that demonstrate proven antibacterial efficacy.

The technique for performing pulpectomies in primary teeth has evolved considerably in recent years. Rotary instrumentation systems have demonstrated significantly shorter instrumentation and obturation times, reduced postoperative discomfort, and enhanced obturation quality, notably through a decrease in the number of voids^6^ Nevertheless, no significant differences have been reported in clinical or radiographic outcomes at 3- or 6-month follow-ups, nor in children’s behavioural responses during treatment^7^. Volumetric studies have shown that rotary files remove less dentin compared to manual files, resulting in reduced apical transportation, higher cantering ability, better preservation of the original canal curvature, and decreased incidence of dentinal microcracks^8,9^. Nickel–titanium (NiTi) files have further improved file flexibility and resistance to cyclic fatigue. Given that the cross-sectional design of NiTi files exerts a greater influence on torsional and bending stress during root canal instrumentation than the instrument’s diameter or size, it is essential to have a thorough understanding of the specific instruments employed^10^. Regarding the type of motion, evidence suggests that reciprocating rotary systems exhibit superior resistance to cyclic fatigue compared to continuous rotation systems, particularly when tested in curved canals^11^.

A wide range of filling materials has been proposed for pulpectomy procedures in primary teeth, including iodoform-based pastes, zinc oxide-eugenol (ZOE) cement, Aloe vera, propolis, and calcium hydroxide (CaOH2) formulations. Nevertheless, there is currently no consensus regarding the optimal filling material for use in primary dentition^12^. ZOE has demonstrated superior antimicrobial efficacy compared to CaOH2-based combinations with other agents such as chlorhexidine, ZOE itself, or iodoform paste^13^. Based on current evidence, CaOH2/iodoform paste appears to be the most suitable obturating material for primary teeth approaching exfoliation. In contrast, for primary teeth expected to remain in the oral cavity for extended periods (more than 18 months), ZOE or ZOE/iodoform combined with CaOH2 are considered the materials of choice^14,15^.

The primary cause of root canal treatment failure is recurrent infection due to residual bacterial contamination and necrotic tissue that was not adequately removed during the initial procedure. This justifies the need for retreatment, which involves the removal of the existing obturation material and re-instrumentation of the infected root canal walls. However, complete removal of root canal filling material is particularly challenging in primary teeth due to their complex anatomy, including narrow, curved canals and fine apical termini^16^. The need for pulpal retreatment is considered a clinical failure of pulpectomy^17^. Although the reported success rate of pulpectomies at 9 months ranges from 85% to 95.7%, depending on the obturating material used^18^, this rate tends to decline over time. At 18 months, success rates drop to 71.4–96.2% in clinical evaluations and 53.6–92.5% in radiographic assessments^19^. A longitudinal study with a 5-year follow-up revealed a pulpectomy success rate of 81.4–87.4%, identifying preoperative radiographic radiolucency as the only factor significantly associated with treatment failure. Other variables such as patient sex, age, dental arch, tooth type, anaesthesia type, obturation material, and final restoration showed no significant influence^17^. Regarding the relationship between obturation material and pulpectomy failure, underfilled root canals are significantly more prone to failure than those that were adequately filled. Another critical variable is the presence of voids or air bubbles within the obturation material, which may lead to fissures and retention of microorganisms and toxins. It is estimated that only 50.78% of primary teeth are adequately filled after pulpectomy procedures, with underfilling occurring in 35.93% of cases across canal systems^20^ The location and size of these voids depend on the type, viscosity, and consistency of the material used, as well as the application technique and operator experience. Although rotary files systems do not influence voids presence when filling, as stated by Kakoienejad et al.^21^, there is no consensus in the relation between filling method and voids presence. According to a recent systematic review^22^, the lowest risk of overfilling was observed when using ZOE introduced with a plugger-type applicator and cotton pellets. Nevertheless, plugger technique shows the most total volume of voids^23^. Absence of voids in filling material is more frequently found when obturating with systems as NaviTip double side port (Ultradent Products, Inc., United States)^20^ with past injection with the most scores of voids presence. Besides, Pastinject showed similar results in total voids presence, with higher obturation rates^23–25^. However, differences in total number of voids is only found in the coronal third in the canals.

Chen et al.^19^ evaluated the prevalence of teeth in which the root remained intact while the filling material underwent resorption, finding that in cases filled with CaOH2-iodoform paste, material resorption occurred in 48.2% of cases at 6 months, 25% at 12 months, and 28.6% at 18 months, significantly higher than with other filling materials. This demonstrated a statistically significant correlation between the rate of material resorption and radiographic failure of the pulpectomy. Although ZOE paste exhibits lower resorption rates as a root canal filling material, its extruded material beyond the periapical region showed limited resorption (71.4%, 66.7%, and 66.7% at 6, 12, and 18 months, respectively), and its resorption was slower than that of the dental roots themselves (31.4% and 39.2% at 12 and 18 months). This slow resorption may trigger a foreign body reaction, discouraging its use in certain clinical scenarios. Besides, teeth treated with CaOH2 that required retreatment ultimately had to be extracted, likely due to the material’s potential to induce root resorption^26^. However, despite 30% of teeth treated with CaOH2 and iodoform paste showing resorption of the filling material, no clinical or radiographic signs of treatment failure were observed. Therefore, in many cases it is necessary to refill the canal system, giving the tooth greater longevity when necessary, especially in cases of agenesis of permanent successors, pulp pathology in second deciduous molars in the absence of emergence of the first permanent molars, or in those cases where the placement of a space maintainer is not recommended (e.g., children with special needs, prevention of malocclusions or harmful oral habits, or promotion of development of adequate oral functions.).

As stated previously, when pulpal treatment fails in absence of clinical or radiographic pathology, is necessary the removal of the root canal filling material to perform retreatment, in order to prevent premature primary tooth loss. Studies conducted on permanent teeth have shown that the removal phase of paste-type root canal filling materials can be carried out using various techniques, including manual instruments, rotary files, dedicated retreatment files, ultrasonic activation, Gates Glidden burs, or chemical solvents. However, none of these methods have proven capable of completely eliminating obturation material from the root canals of primary teeth^27^. Akman et al.^28^ compared different irrigation activation protocols for the removal of modified triple antibiotic paste from root canals. Their findings indicated that passive ultrasonic irrigation activation improved cleaning efficacy in the coronal third of the canal. Due to the still high prevalence of caries in primary teeth and the potential damage caused by premature tooth loss, it is of great importance for paediatric dentists to understand the capacity of canal systems to remove and refill tooth canals. Improvements in this area of ​​knowledge would allow for more accurate and individualized decisions in cases where preserving primary teeth in the dental arch is a priority, providing conservative therapeutic alternatives to tooth extraction. The main aim was to analyse the efficacy of Endogal Kids^®^ rotary files in removing root canal filling material (CaOH2-iodoform paste) from primary teeth assessed by micro-computed tomography (µCT). The evaluation of the canal wall wear following retreatment was established as a second objective.

## Materials and methods

### Study design

A cross-sectional, analytical, controlled, and randomized study was conducted, involving in vitro experimentation on primary teeth obtained from patients who attended the Paediatric Dentistry Master’s Clinic at the University Specialty Clinic of Alfonso X El Sabio University (UAX) in Madrid, Spain. This manuscript was prepared in accordance with the “Reporting stAndards for research in PedIatric Dentistry” (RAPID), following the checklist for the ‘Pulp Therapy’ theme^29^.

### Ethics statement

The study was conducted in accordance with the ethical principles outlined in the Declaration of Helsinki (2013). The study protocol was approved by the Bioethics Committee of Alfonso X El Sabio University (Resolution No. 2023_05/213, May 2023). Informed consent was obtained from all participants, or from their parents and/or legal guardians in the case of subjects under 16 years of age. Additionally, children over 12 years of age also provided verbal assent prior to the start of the study.

### Participants and sample size

A priori sample size calculation was performed with G*Power software (version 3.1.9.7., Dusseldorf, Germany)^30,31^, determining that 12 teeth would be required to achieve statistical significance for a related-samples analysis involving three measurements, assuming a large effect size (0.45), a 5% alpha error, and 80% statistical power. Extracted primary teeth were included if removed for orthodontic or restorative reasons, with no previous root canal filling material and no evidence of root canal resorption. Teeth presenting root resorption detected during instrumentation or perforations identified during µCT analysis were excluded. After the exclusion of one tooth following the initial µCT analysis, a total of 10 primary teeth were selected from those collected and deemed suitable for the study within a two-month period.

### Outcomes

Tooth type (primary molar or incisor) was considered as a variable. Canal void volume (CV) and obturation material volume (MV) were measured in mm³^32^. CV was assessed at three time points: time point 1 (CV-1), measured in the first µCT, corresponded to the volume of the instrumented but empty root canal; time point 2 (CV-2), measured in the second µCT, represented the void volume after obturation; and time point 3 (CV-3), measured in the third µCT, reflected the void volume after removal of the obturation material. MV was measured only in the second and third µCT scans, representing the volume of obturation material present (MV-2) and the residual material volume after removal by instrumentation (MV-3). Based on these two variables, a theoretical variable termed total volume (TV) was created, corresponding to the sum of CV and MV at each time point. Thus, the initial total volume (TV-1) equalled CV-1, while the intermediate (TV-2) and final total volumes (TV-3) were the sum of the respective CV and MV values.

Derived variables were created: root canal void increase (ΔCV) and obturation material increase (ΔMV). ΔCV was calculated as the difference between the CV measurements, both in mm³ and as a percentage relative to the previous measurement. Similarly, ΔMV was calculated as the difference between MV measurements, in mm³ and as a percentage relative to the preceding value. Additionally, the variable ‘filling removal volume’ (RV) was calculated.

### Pulpectomy procedure

Before instrumentation, the canals of all teeth were evaluated to confirm canal patency and exclude teeth with anatomical alterations. Preoperative digital diagnostic radiographs were obtained by paralleling a radiographic film with the dental axis (X-Mind^®^ Unity, Acteon^®^ group, Lyon, France) and NemoStudio software (version 24.0.0.3, Nemotec SL, Leganés, Spain, https://nemotec.com/nemostudio/es/). Once confirmed the suitability, the specimens were stored at room temperature with 100% humidity in a 10% formalin solution until use, to prevent dehydration.

All endodontic procedures were performed by the same operator experienced in pulp treatments in children using rotary files in primary dentition. The teeth were placed in polyvinyl siloxane silicone (Express-2 Putty Soft, M ESPE, Saint Paul, MN, EE. UU.) to maintain a reproducible orientation across all procedures. In order to work in a standardized and reproducible manner, access cavities to the root canals were prepared using the technique described by Rover et al.^33^. Canal patency and working length were determined using a size 12 stainless steel pre-K file (Endogal, Sarria, España). The WL was measured as 0.5 mm shorter than the canal length from the crown to the anatomical apex prior to the rotary file instrumentation.

The root canals were instrumented using the Endogal Kids^®^ system (Endogal, Sarria, Spain), following the Endogal Kids protocol, operated by the wireless Endogal motor with continuous rotation at 350 rpm and a torque level of 4 Ncm. Each file was used in three root canals and then discarded. EK1 files (25 mm diameter with 4% taper) were used for narrow canals, which included the mandibular mesiobuccal and maxillar distobuccal canals. For medium-sized canals, EK2 files (25 mm diameter with 6% taper) were used, corresponding to the mandibular distal and maxillar mesiobuccal canals. For wide canals, corresponding to the palatal canals, EK3 files (30 mm diameter with 4% taper) were utilized. Finally, EK4 files (40 mm diameter with 4% taper) were used for anterior teeth canals.

Irrigation was performed with 5 mL of 5% sodium hypochlorite solution (Braun, Barcelona, Spain) between each filing movement (manual and rotary), using a sterile NaviTip™ 31-gauge lateral outlet needle with double side port irrigation (Ultradent Products Inc., South Jordan, UT, USA) between each file change. The canals were dried with absorbent paper points (Dentsply Maillefer) and the first µCT was conducted.

Secondly, root canals were obturated with CaOH2 and iodoform paste, Forendo™ Paste (Pulpdent Corporation, Watertown, USA). Access cavities were temporarily restored with Cavit^®^ (3 M Espe, Seefeld, Germany) to prevent filling material movement, and periapical parallel radiographs (X-Mind^®^ Unity, Acteon^®^ group, Lyon, France) were taken for follow-up to assess the quality of the filling and sealing, and once validated, the specimens were stored at room temperature with 100% humidity in a 10% formalin solution until use. The samples were sent for the second µCT scan.

In a third phase, the canal systems were retreated to remove the filling material using continuous counterclockwise rotation, following the same irrigation and drying protocol as before. Finally, the teeth were stored at room temperature with 100% relative humidity. Finally, the last µCT scan was performed.

### µCT analysis procedure

µCT scans were performed at three time points: (1) after canal instrumentation with rotary files (µCT-1), (2) after root canal filling (µCT-2), and (3) after removal of the filling material through retreatment (µCT-3). All µCT scans were performed using the high-resolution 3D X-ray microscopy scanner SkyScan Bruker^®^ 1272 CMOS (Bruker Corporation, Massachusetts, USA). For all cases, the scanning parameters were: 12 μm resolution, 0.3º rotation step, two frames per scan point (frame averaging), 180º sample rotation, and a 0.25 mm aluminium filter. Measurements below 2 pixels were not feasible; therefore, dentin wear < 24 μm was not detectable. In all 3D scans, the teeth were positioned with the roots facing upward.

After scanning the samples, 3D reconstruction was performed using Bruker^®^’s ‘NRecon’ software (version V2, Bruker Corporation, Massachusetts, USA). The data were processed using the open-source software ‘Data Viewer’ for slice-by-slice inspection of 3D volumes and 2D/3D image registration, with the objective of aligning the images in different orthogonal planes in preparation for data acquisition. Image quantification, analysis, and processing were carried out using Bruker^®^’s ‘CTAn’ software (version 1.2021, Bruker Corporation, Massachusetts, USA), defining a constant Volume of Interest (VOI) for each root of every evaluated tooth. The VOI extended from the cemento-enamel junction of the crown to the apex of each root canal. The measurement was conducted by determining the required grayscale range for recognising the filling material and the root canal under study through a density histogram, utilising a threshold method to obtain a binary image composed solely of black and white pixels.

**Fig. 1:**
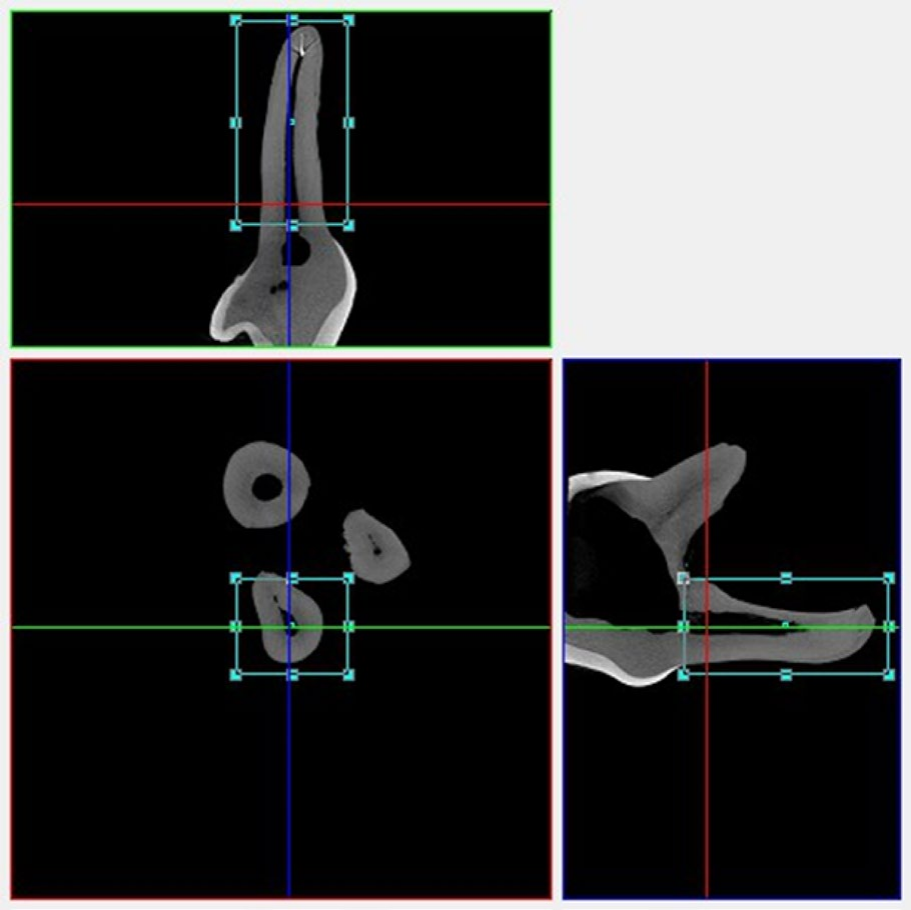
2D reconstruction planes of sample 6 (palatal root of a second upper primary molar) evaluated in µCT-1, showing details of the three orthogonal planes and the selected Volume of Interest (VOI).

**Fig. 2:**
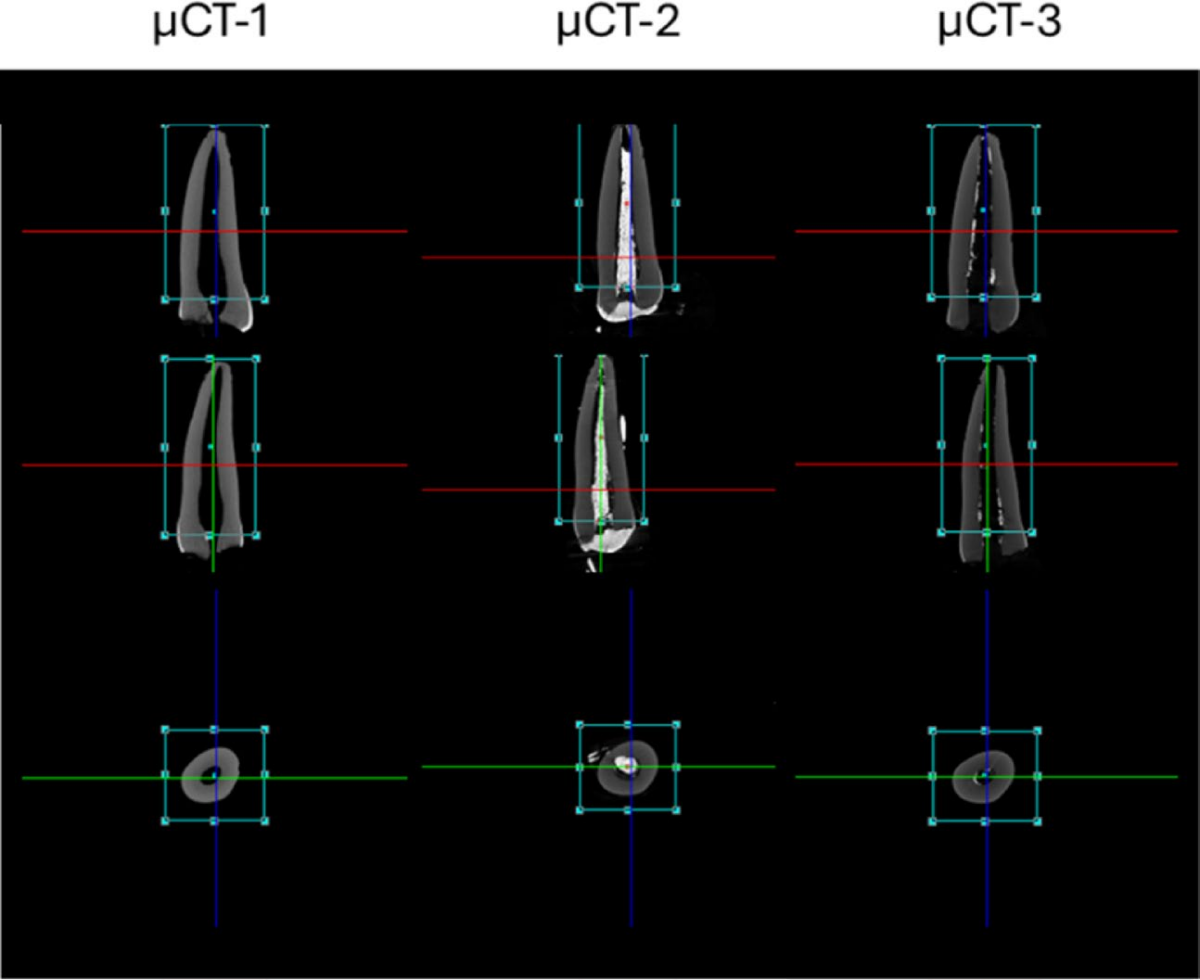
2D reconstruction planes of sample 9 (incisor) evaluated in the three µCT scans, showing details of the three orthogonal planes and the selected Volume of Interest (VOI).

Once the VOI was established, the images were segmented using thresholding based on the 255-gray level histogram obtained during reconstruction, allowing differentiation in each scanned image between the empty internal volume of the dental root and the volume occupied by the inorganic phase (Fig. 1). To ensure methodological consistency, all samples were evaluated following a standardized protocol, aligninf the same VOI in the three µCT scans (Fig. 2).

### Blinding

All dental preparations were performed by the same operator, a paediatric dentist with expertise in root canal instrumentation in primary teeth. As there was only one study group, blinding of the samples was not necessary. The µCT scans were performed and analysed by an external service, which was unaware of the nature of the study, thus ensuring blinding to potential measurement outcomes. Additionally, the statistician was independent from the study, preventing bias in the interpretation of the results.

### Statistical method

Statistical analysis was performed using the IBM^®^ SPSS^®^ software (version 29.0, Armonk, NY, USA). Means and standard deviations were calculated to describe the study variables. The Shapiro–Wilk test was used to assess normality. Differences between the two tooth groups were analysed using the Student’s t-test. A repeated measures analysis was conducted using the Friedman test for related samples in CV, MV, and TV, with Bonferroni post-hoc correction. All tests were performed with a 95% significance level (*p* ≤ 0.05) and two-tailed significance.

## Results

### Data description

A total of eight first primary molars (seven uppers and one lower) and three incisors were collected. CV was initially analysed (Table 1). It was assessed whether CV differed between incisors and molars, revealing that the volume was significantly greater in incisors (11.56 ± 4.74 mm³) compared to molars (2.71 ± 1.28 mm³) (T-test *p* = 0.001).

**Table 1:** Description of canal volume (CV) in the study sample across the three evaluations performed.

Tooth type	Sample	Location	Root	CV (mm^3^)		
				µCT-1	µCT-2	µCT-3
Molar	1	Upper	Palatal	4.835	2.103	3.535
	2	Upper	Palatal	1.936	1.035	1.807
	3	Upper	Palatal	1.686	0.574	1.543
	4	Upper	Distobuccal	1.458	0.432	1.373
	5	Upper	Palatal	2.624	0.791	1.573
	6	Upper	Palatal	2.167	2.027	2.136
	7	Upper	Distobuccal	2.457	0.470	0.835
	8	Lower	Mesial	4.553	1.583	3.069
Incisor	9	Upper	–	9.129	5.322	7.577
	10	Upper	–	8.524	5.112	6.754
	11	Upper	–	17.021	13.756	9.786
MEAN				5.126	3.019	3.635
SD				4.765	3.961	3.010

Changes in TV (Table 2) along the three µCT scans were analysed, showing minimal variation throughout the experimental phases. A mean increase of 4% (range: − 39% to 53%) was observed after the first instrumentation (µCT‑1 to µCT‑2), followed by a further 1% increase (range: − 29% to 23%) after retreatment (µCT‑2 to µCT‑3). Overall, the cumulative change in TV from baseline to the final scan represented a net increase of 1% (range: − 27% to 37%), indicating that rotary re‑instrumentation produced only slight modifications in the TV. Across the three µCT comparisons, molars showed wide percentage variability in TV, with changes ranging from − 39% to + 53% after obturation (ΔTV 2–1), − 22% to + 23% after retreatment (ΔTV 3–2), and cumulative changes of − 27% to + 37% from baseline to the final scan (ΔTV 3–1). In contrast, incisors presented more consistent reductions: − 17% to + 21% for ΔTV 2–1, − 29% to − 17% for ΔTV 3–2, and overall decreases of − 14% to − 8% for ΔTV 3–1.

**Table 2:** Theoretical data of total volume (TV) at different µCT evaluations.

Sample	Tooth type	TV (mm^3^)			ΔTV (mm^3^)			ΔTV (%)		
		µCT-1	µCT-2	µCT-3	2 − 1	3 − 2	3 − 1	(2 − 1)/1	(3 − 2)/2	(3 − 1)/1
1	Molar	4.835	4.457	5.075	– 0.379	0.619	0.240	– 8%	14%	5%
2	Molar	1.936	1.898	2.335	– 0.038	0.437	0.399	– 2%	23%	21%
3	Molar	1.686	2.048	2.307	0.361	0.259	0.621	21%	13%	37%
4	Molar	1.458	1.800	1.599	0.342	– 0.201	0.141	23%	-11%	10%
5	Molar	2.624	2.026	2.481	– 0.598	0.456	– 0.142	-23%	22%	-5%
6	Molar	2.167	3.305	2.570	1.138	-0.735	0.403	53%	-22%	19%
7	Molar	2.457	1.500	1.793	– 0.956	0.292	– 0.664	– 39%	19%	– 27%
8	Molar	4.553	4.983	4.229	0.430	-0.754	-0.324	9%	– 15%	– 7%
9	Incisor	9.129	7.532	8.365	-1.597	0.833	– 0.764	– 17%	11%	– 8%
10	Incisor	8.524	8.927	7.399	0.404	– 1.529	– 1.125	5%	– 17%	– 13%
11	Incisor	17.021	20.673	14.581	3.652	– 6.091	– 2.440	21%	– 29%	– 14%
MEAN		5.126	5.377	4.794	0.251	– 0.583	– 0.332	4%	1%	1%
SD		4.765	5.646	3.968	1.360	1.962	0.891	26%	20%	19%

Regarding filling material volume (MV) (Table 3), a substantial reduction was detected between µCT‑2 and µCT‑3. On average, 52% of the filling material was removed during retreatment, with removal values ranging from 7% to 83%. Regarding tooth type, incisors demonstrated markedly higher removal between µCT‑2 and µCT‑3, frequently exceeding 60% of filling material, whereas molars showed more heterogeneous reductions (7–83%).

**Table 3:** Theoretical data of filling material volume (MV) at different µCT evaluations.

Sample	Tooth type	MV (mm^3^)			ΔMV (mm^3^)			ΔMV (%)		
		µCT-1	µCT-2	µCT-3	2 − 1	3 − 2	3 − 1	(2 − 1)/1	(3 − 2)/2	(3 − 1)/1
1	Molar	–	2.354	1.541	–	– 0.8129	–	–	– 53%	–
2	Molar	–	0.863	0.528	–	– 0.33477	–	–	– 39%	–
3	Molar	–	1.474	0.764	–	– 0.71015	–	–	– 48%	–
4	Molar	–	1.369	0.226	–	– 1.14277	–	–	– 83%	–
5	Molar	–	1.235	0.909	–	– 0.32588	–	–	– 26%	–
6	Molar	–	1.278	0.434	–	– 0.84418	–	–	– 66%	–
7	Molar	–	1.031	0.958	–	– 0.07276	–	–	– 7%	–
8	Molar	–	3.400	1.160	–	– 2.2402	–	–	– 66%	–
9	Incisor	–	2.210	0.789	–	– 1.42156	–	–	– 64%	–
10	Incisor	–	3.816	0.644	–	– 3.17142	–	–	– 83%	–
11	Incisor	–	6.917	4.795	–	– 2.1217	–	–	– 31%	–
MEAN			2.359	1.159		– 1.200			– 52%	
SD			1.794	1.258		0.958			24%	

Normality of the volumetric variables was assessed, revealing that only ΔTV (2 − 1), ΔTV (3 − 1), ΔMV, and all percentage measurements met normality criteria (Shapiro-Wilk *p* > 0.05). However, when categorized by tooth type, all variables satisfied normality assumptions (Shapiro-Wilk *p* > 0.05).

### Analysis of the influence of tooth type on the study variables

**Fig. 3:**
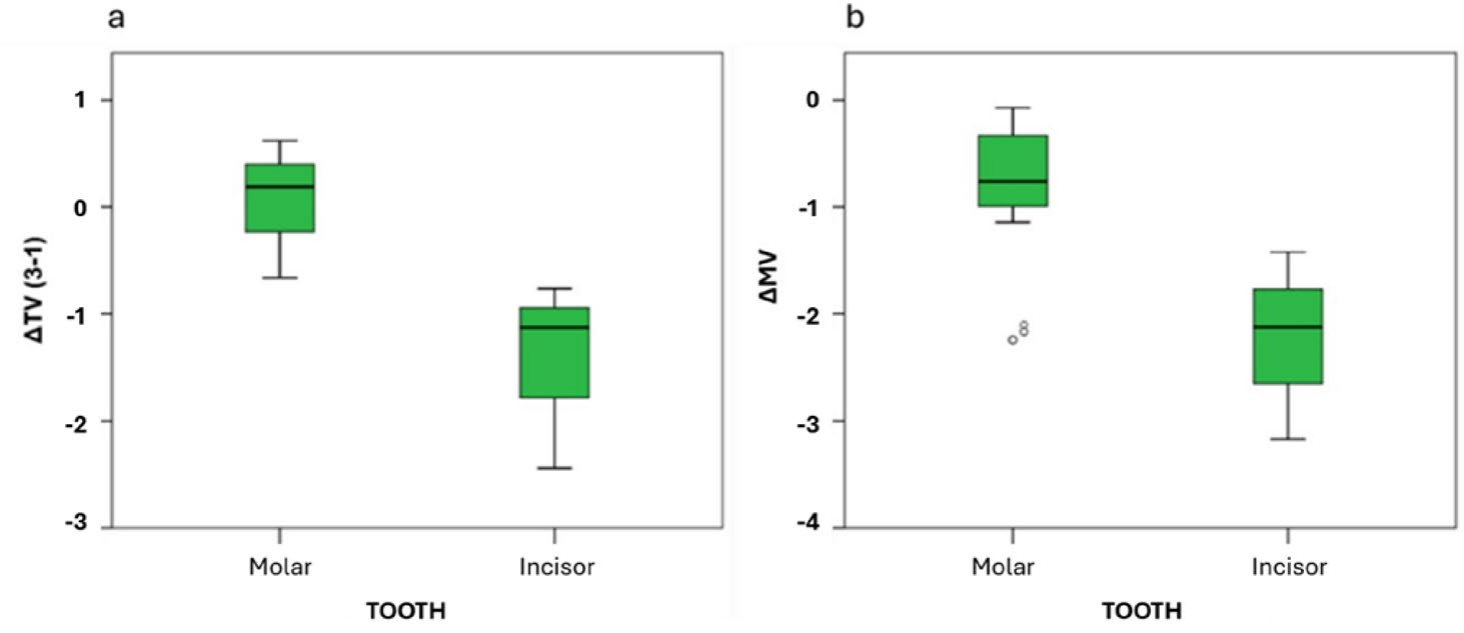
Boxplots showing statistically significant differences in ΔTV (3 − 1) (**a**) and ΔMV (**b**) outcomes.

The influence of tooth type on the volumetric increase of TV and MV was analysed (Table 4). No significant differences were observed in the early changes in total volume (∆TV 2–1) or in the subsequent interval (∆TV 3–2). Significant differences were obtained in ∆TV and ∆MV (Fig. 3). Results indicate that significant differences were only found in the cumulative change from baseline to the final scan (∆TV 3–1) showed a statistically significant difference between tooth types (*p* = 0.003), with molars exhibiting slight increases in TV, while incisors showed net reductions. In relation to ∆MV, incisors showed a significantly higher absolute reduction compared with molars (*p* = 0.017), consistent with more efficient paste removal in anterior teeth.

**Table 4:** Descriptive and inferential analysis of volumetric and percentage increases in total volume (TV) and obturation material volume (MV). Significant results *p* ≤ 0.05.

Outcome	Unit	Tooth	N	Mean	Standard deviation	T-test	95% Confidence Interval	
						P value	Lower limit	Upper limit
ΔTV (2 − 1)	mm^3^	Molar	8	0.0375	0.66897	0.425	– 2.89736	1.33302
		Incisor	3	0.8197	2.64907			
ΔTV (3 − 2)	mm^3^	Molar	8	0.0466	0.54329	0.080	– 0.33597	4.95388
		Incisor	3	-2.2623	3.51977			
ΔTV (3 − 1)	mm^3^	Molar	8	0.0843	0.42995	0.003*	0.66541	2.38909
		Incisor	3	-1.4430	0.88209			
ΔMV	mm^3^	Molar	8	-0.8105	0.67306	0.017*	0.31841	2.53715
		Incisor	3	-2.2382	0.88073			
ΔTV (2 − 1)	%	Molar	8	4.25	28.868	0.947	– 40.102	42.602
		Incisor	3	3.00	19.079			
ΔTV(3 − 2)	%	Molar	8	5.38	18.275	0.213	– 11.748	45.832
		Incisor	3	-11.67	20.526			
ΔTV(3 − 1)	%	Molar	8	6.63	19.813	0.158	– 8.569	45.152
		Incisor	3	-11.67	3.215			
ΔMV	%	Molar	8	-48.50	24.325	0.535	– 27.118	48.784
		Incisor	3	-59.33	26.312			

### Longitudinal analysis of the variation in study variable

**Fig. 4:**
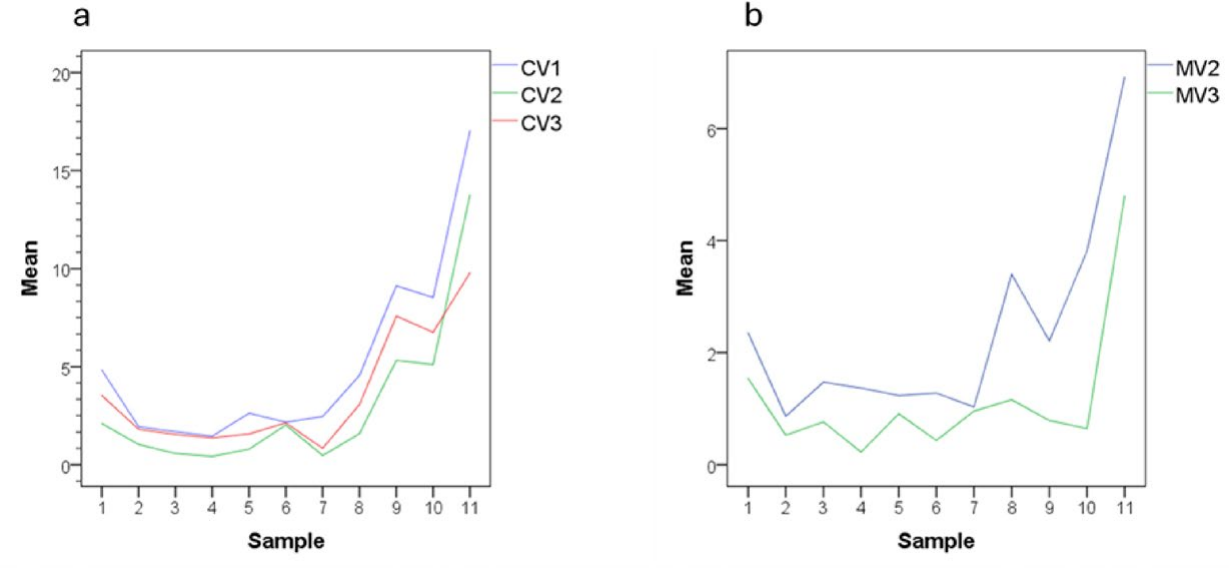
Line diagram illustrating statistically significative differences in CV (**a**) and MV (**b**) in µCT repeated measures analysis.

A repeated measures analysis was performed to assess whether the study variables (CV, MV, and TV) differed across the µCT time points (Table 5; Fig. 4). Significant differences were found for CV and MV, but not for TV. Post-hoc tests indicated that the CV was significantly greater at the µCT-1 (pre-obturation) compared to µCT-2 (post-obturation) (*p* < 0.001) and µCT-3 (post-removal of obturation material) (*p* = 0.032) scans, with no significant difference between µCT-2 and µCT-3. Additionally, MV was significantly higher before removal of the filling material (*p* = 0.003). No significant differences were observed in the total canal volume (TV) across the three measurements (Friedman test, *p* = 1).

**Table 5:** Significance values from the Friedman test and post-hoc analysis for the variables CV, MV, and TV. Significant results *p* ≤ 0.05.

Outcome	Friedman test	µCT	Bonferroni test
	*P* value		*P* value
CV	< 0.001*	2–3	0.165
		2 − 1	< 0.001*
		3 − 1	0.032*
MV	0.003*	3 − 2	-
TV	1	2–3	-
		2 − 1	-
		3 − 1	-

## Discussion

This pilot study aimed to evaluate the volumetric changes in root canal space and the removal efficacy of an iodoform-based paste in primary teeth using rotary instrumentation, assessed through high-resolution µCT. The results provide valuable insights into the behaviour of obturation materials and the influence of tooth type on canal morphology and paste removal. Our findings align with previous µCT-based investigations showing that rotary instrumentation produces minimal dentin removal in primary teeth and that complete elimination of paste-type materials remains challenging, particularly in multi-rooted molars. Similar to reports by Smutkeeree et al.^34^, Marques et al.^35^, and Liu et al.^36^, our data confirm that anatomical complexity strongly influences the amount of residual material. Moreover, the high removal efficiency observed in incisors is consistent with studies indicating that straight, wider canals facilitate irrigant penetration and file contact during retreatment.

Several studies have investigated the removal efficacy of various intracanal pastes in permanent teeth using different irrigation and instrumentation systems^37–46^. The majority of these studies have focused on the removal of CaOH_2_ as the intracanal medicament^38,40,41,43–45^, with the exception of Turkaydin et al.^37^, who evaluated the removal of both CaOH_2_ and a compound paste composed of CaOH_2_, iodoform, and p-chlorophenol, and Liu et al.^36^, who tested CaOH_2_ mixed with barium sulphate in different formulations. Additionally, Shakapuram et al.^42^. assessed the removal of three different intracanal medicaments (Triple Antibiotic paste, Odontopaste, and Metapex) using passive ultrasonic irrigation (PUI). Regarding sample types, Kurt et al.^46^ used 80 resin monoblocs, while Marques et al.^35^ employed 120 artificial primary teeth with manual instrumentation in incisors and molars. Smutkeeree et al.^34^ used 72 extracted primary molars. Our sample size is significantly lower, with only 11 extracted teeth.

While numerous studies have analysed instrumentation efficacy, dentin preservation, and obturation quality in primary teeth, very few have quantified volumetric behaviour across sequential treatment phases. Most existing µCT studies focus on either pre- and post-instrumentation or post-obturation alone, without evaluating the retreatment phase. By contrast, our three-time-point analysis provides a more detailed characterization of changes occurring at each stage of the endodontic workflow, highlighting aspects of canal morphology and paste behaviour that have not been previously quantified in primary teeth. To our knowledge, this is the first study to assess CaOH₂–iodoform paste retreatment in primary teeth using a triple µCT evaluation protocol. This approach provides unprecedented temporal resolution to quantify how obturation and retreatment affect canal volume and residual filling material.

The evaluation of obturation quality in pulpectomy procedures has evolved considerably in recent years. Intraoral digital radiography remains the most commonly used method in clinical practice due to its accessibility and low cost, as reported by Memarpour et al.^47^ and Khadilkar et al.^48^. However, being 2D technique, it presents significant limitations in assessing the 3Dseal of the filling material, particularly in detecting internal voids or visualizing overlapping anatomical structures. CBCT (Cone Beam Computed Tomography) offers improved volumetric visualization, yet it is constrained by relatively low spatial resolution and the presence of imaging artifacts, such as the cone beam hardening effect, which can simulate cracks or voids that do not correspond to actual defects^49–51^. In this context, µCT has emerged as the method of choice for in vitro studies due to its high precision, non-invasive nature, and ability to perform repeated scans without damaging the sample^50,52–54^. Unlike CBCT, µCT’s smaller field of view minimizes artifacts by avoiding the traversal of dense anatomical structures or soft tissues^52,55^. Authors such as Jung et al.^53^ and Singh et al.^56^ have highlighted its utility in comparing pre- and post-obturation volumes, allowing for highly reliable calculations of filling percentages. This level of precision far exceeds that of earlier techniques such as serial histological sectioning or conventional radiography, which lack 3D quantification and compromise sample integrity. Various methodologies have been employed to assess endodontic file wear, dentinal wall alterations, and the removal of root canal filling materials during retreatment procedures. These include CBCT, SEM (scanning electron microscope), stereomicroscopy, and µCT. Among these, µCT is widely regarded as the gold standard for evaluating root canal obturation removal due to its high-resolution, 3Dimaging capabilities, and its non-destructive nature, which minimizes the risk of sample fracture or measurement artifacts. Most studies analysing root canal morphology and filling material removal using µCT employ voxel sizes around 11.6 μm, although resolutions as fine as 5–6 μm have been reported^57^. For optimal canal morphology analysis, a voxel size of 76 μm has been suggested^58^. In our study, a resolution of 12 μm was used, allowing for high-precision evaluation of canal volume, dentin wear, and residual obturation material.

The initial analysis of CV revealed a statistically significant difference between tooth types, with incisors presenting a significantly greater mean canal volume compared to molars. This finding is consistent with the anatomical differences between anterior and posterior primary teeth, where incisors typically exhibit larger and straighter canals, facilitating greater volumetric capacity. Regarding TV, the mean increase after the first instrumentation was 4%, and 1% after the second instrumentation. However, the overall increase from baseline to the final scan was only 1% (mean = -0.332 ± 0.891 mm), revealing that tooth retreatment does not cause over-wear of the root canal system. These modest changes suggest that rotary instrumentation in primary teeth may be conservative in terms of dentin removal, which is clinically relevant for preserving tooth structure in paediatric patients. Notably, no statistically significant differences in TV were observed across the three µCT time points, indicating that the instrumentation protocol did not significantly alter the total canal space. In contrast, significant differences were observed in CV and MV across time points. CV was significantly reduced after canal filling compared to the pre-filling scan, and partially restored after paste removal, although not to baseline levels. This suggests that while rotary instrumentation was effective in removing a substantial portion of the filling material, complete restoration of the original canal space was not achieved. The mean volume of filling material removed was 52%, with a wide range from 7% to 83%. This variability may reflect differences in canal anatomy, paste adaptation, and the limitations of rotary files in accessing irregularities or resorption areas common in primary teeth. Interestingly, the removal efficacy was significantly influenced by tooth type. Incisors showed a significantly higher absolute ΔMV compared to molars, with percentages of filling material removal of 59.33% and 48.50%, respectively, indicating more effective paste removal in anterior teeth. This may be attributed to their simpler canal morphology and greater initial volume, which facilitates better irrigant penetration and file manoeuvrability. Conversely, the volumetric increase in total space from pre-filling µCT to final µCT was significantly greater in molars (6.63%) than in incisors (-11.67%), suggesting that molars may undergo more structural alteration during instrumentation, possibly due to their more complex anatomy and the need for more aggressive shaping to access all canal areas. The marked variability in molars contrasts with the more predictable behaviour observed in incisors. This reinforces the need for tooth-type-specific retreatment strategies, as molars, particularly those with physiological resorption, may retain more material and undergo greater morphological fluctuation during instrumentation.

Numerous studies have assessed the effectiveness of different irrigation systems for removing intracanal CaOH_2_ in permanent teeth, including EndoActivator (sonic), CanalBrush (motor-driven brush), passive ultrasonic irrigation (PUI), EndoVac (negative pressure), ProUltra (continuous ultrasonic), conventional syringe irrigation, and RinsEndo (hydrodynamic)^38,40–46^. Despite this variety, there is no consensus on the most effective method, and all studies agree that none of the systems can completely eliminate the medicament. Turkaydin et al.^37^ found that Calcipast Forte (CaOH_2_ with iodoform and p-chlorophenol) was more difficult to remove than pure CaOH_2_. Liu et al.^36^ tested several irrigations systems, and found that pure CaOH_2_ and its mixture with barium sulfate (8:1) were removed more efficiently. Shakapuram et al.^42^ reported that Odontopaste had the lowest residual scores, while Triple Antibiotic Paste and Metapex showed higher retention. EDTA (ethylenediaminetetraacetic acid) has been shown to chemically enhance CaOH2 removal^59–61^. Consistent with prior literature^62–64^, more residual CaOH2 is typically found in the apical third, possibly due to apical compaction during placement or removal.

A recent systematic^65^ review evaluated whether rotary or manual instrumentation better preserves dentin thickness. The majority of studies concluded that rotary instrumentation results in greater remaining dentine thickness, suggesting that it is less aggressive and better preserves tooth structure.

Although none of the published studies in primary teeth replicate our methodology, several have focused on evaluating obturation quality and filling capacity of different pastes^46–48^. There is still no consensus on the ideal material for obturating primary root canals. Kurt et al.^46^ compared CaOH2 pastes containing either iodoform or barium sulfate at different temperatures. They found that the iodoform-containing group exhibited significantly higher radiopacity than the barium sulfate group. Additionally, they observed that higher temperatures improved the filling ability, with fewer unfilled areas and better adaptation. Marques et al.^35^ tested an experimental paste against Vitapex, analyzing obturation quality. Experimental paste showed a significantly lower failure rate (12.5%) compared to Vitapex (31.5%). Experimental paste also demonstrated superior flowability, radiopacity, pH, and filling capacity. Moreover, the use of a fine-tip syringe was significantly more effective than Lentulo spiral carriers. Among tooth types, molars showed the highest failure rate in obturation quality. Smutkeeree et al.^34^ evaluated the filling quality of CaOH2 using both syringe and Lentulo spiral techniques, reporting no statistically significant differences in the percentage of filled area between materials or techniques. Their analysis, based on 2D projections, estimated a mean filling percentage of 83.57% buccolingually and 84.71% mesiodistally. In contrast, our study employed 3D µCT to assess obturation and removal, providing a more comprehensive and accurate evaluation of canal filling and residual material. Furthermore, the authors assessed obturation quality using criteria such as completeness of obturation, presence of air inclusions, and apical fill quality, finding that 50% of the Lentulo group achieved adequate apical fill, but no significant differences were observed among groups. Our study, by contrast, quantified residual material volumetrically, offering a more objective and reproducible method for evaluating obturation quality and removal efficacy.

A recent systematic review^66^ aimed to determine which materials demonstrated higher clinical success rates compared to ZOE. The results indicated that ZOE combined with CaOH2 and iodoform showed improved clinical and radiographic success rates, a resorption rate similar to that of the roots, faster resorption of extruded particles, and a greater reduction in the size of preoperative interradicular radiolucencies. However, none of the materials evaluated could be considered ideal for use in primary teeth. Another recent systematic review^12^ evaluated the clinical and radiographic success rates of various obturation materials used in primary teeth. Eleven different materials were assessed, with ZOE being used either as the test material or as the control group in all trials. Conventional materials included Vitapex, Metapex, and Endoflas, while newer materials included zinc oxide (ZnO) with iodoform, MPRCF (Mixed Primary Root Canal Filling), ZnO with ozonated sesame oil, ZnO with Aloe vera extract, ZnO with 10% NaF, and ZnO with propolis. A secondary objective of the review was to assess the quality of obturation. Only two studies evaluated this parameter, and neither reported statistical significance, making it difficult to establish a clear relationship between the obturation material and the quality of the fill. Among the materials, MPRCF—similar in composition to Endoflas—was considered the most promising due to its high success rate. However, the overall level of evidence supporting the superiority of any new material remains low.

Marques et al.^35^ and Kurt et al.^46^ evaluated the filling ability of materials by measuring and summing the unfilled canal areas in square millimetres using 2D radiographs. In contrast, our study employed µCT, providing a more accurate volumetric assessment. Smutkeeree et al.^34^ also estimated the percentage of canal filled with CaOH2 based on canal area, a method similar in concept to ours, although limited to 2D projections. The quality of obturation was assessed by evaluating the radiopacity and internal homogeneity of the materials, as observed in radiographic images. Kurt et al.^46^ and Marques et al.^35^ compared the unfilled canal areas in mm², while Smutkeeree et al.^34^ used different criteria: completeness of obturation, number of air inclusions or filling deficiencies, and the quality of the apical portion of the medication. Smutkeeree et al.^34^ reported that 50% of the group receiving manually mixed CaOH_2_ applied with a Lentulo spiral achieved adequate quality in the apical third. However, no statistically significant differences were found among the four experimental groups in either buccolingual or mesiodistal directions.

From a clinical perspective, understanding the retreatability of CaOH₂–iodoform paste in primary teeth is relevant in specific situations where maintaining the primary tooth is essential, such as in cases of agenesis, delayed eruption of permanent successors, patients with special needs, or when extraction or placement of space maintainers is contraindicated. The minimal dentin alteration observed in our study supports the conservative nature of rotary instrumentation in primary teeth, while the differences found between incisors and molars highlight the need for canal-specific strategies during retreatment. Although retreatment is not universally indicated in paediatric dentistry, these findings provide useful evidence for clinicians facing selected cases in which preservation of the primary tooth is preferred over extraction.

This study presents several limitations that should be acknowledged. One of the main limitations of this study was the inclusion of both curved molar canals and straight, wider incisor canals within the same sample. This anatomical heterogeneity may have introduced bias in the interpretation of the results, particularly in the analysis of canal volume changes and obturation material removal. However, the repeated measures test minimizes this bias, using the same teeth as its own control. Besides, the small sample size, particularly the reduced number of incisors compared with molars, limits the statistical power and may have contributed to the wide variability observed in volumetric outcomes. Additionally, the intra-treatment assessment of obturation quality using digital radiographs, although commonly employed in clinical settings due to their accessibility and low cost, relies on 2D imaging, which is inherently limited in detecting internal voids or evaluating the 3D adaptation of the filling material. This limitation may reduce the sensitivity of detection and lead to underestimation of filling deficiencies. Finally, the in vitro design does not fully replicate clinical conditions, such as physiological resorption patterns, periodontal ligament support, and intraoral moisture, which may influence both instrumentation behaviour and paste removal dynamics in vivo.

A major strength of this study is the use of high‑resolution µCT with a fully standardized scanning and analysis protocol. By applying identical acquisition parameters, a fixed VOI, and consistent image registration across all time points, the study ensured highly reliable and reproducible volumetric measurements, minimizing methodological variability. This approach provides greater precision than traditional 2D radiographic methods typically used in pulpectomy research. To date, no similar studies have been conducted in primary dentition, likely due to the inherent fragility of primary root canals, the presence of apical resorption, and the challenges associated with achieving adequate instrumentation in the apical third. Furthermore, the adhesion of obturation materials to dentinal walls—especially when using fluid pastes rather than thermoplastic materials like gutta-percha—adds complexity to removal procedures. A major strength of this study is the use of high-resolution µCT as the primary evaluation method. This technique allowed for precise, reproducible, and non-destructive 3D analysis of both the removal of obturation material and potential dentin wall wear. Unlike conventional radiographic methods, µCT enables the detection of internal voids and subtle morphological changes that would otherwise go unnoticed. This provides a more accurate and comprehensive understanding of the efficacy of endodontic retreatment procedures in primary teeth. Besides, the repeated measures analysis confirmed that while CV and MV changed significantly over time, TV remained stable. These findings highlight the importance of using high-resolution 3D imaging to detect subtle changes in canal morphology and to quantify the effectiveness of endodontic procedures in primary teeth. The use of µCT in this study allowed for precise volumetric assessment, overcoming the limitations of 2D radiographic methods commonly used in previous studies.

Overall, the findings of this study suggest that while rotary instrumentation is effective in removing a substantial portion of iodoform-based paste in primary teeth, complete removal remains challenging, particularly in molars. Taken together, our findings indicate that retreatment behaviour is strongly influenced by tooth morphology and that rotary instrumentation provides a conservative approach with limited structural impact. These insights may assist clinicians in determining when retreatment is a feasible and biologically sound option in paediatric patients. Although rotary instrumentation appears to be a conservative and effective approach, further are needed to optimize removal protocols and improve clinical outcomes in paediatric endodontics. The use of µCT provides a valuable tool for assessing these outcomes with high precision. Future research should explore the combination of different brands or types of rotary instrumentation with adjunctive techniques such as ultrasonic or laser-activated irrigation to enhance removal efficacy, particularly in anatomically complex canals. Such designs will help determine whether the patterns identified in this pilot study—particularly the tooth-type-dependent behaviour—remain consistent across broader clinical scenarios.

## Conclusions

This pilot study demonstrated that rotary instrumentation in primary teeth is capable of removing a significant proportion of iodoform-based obturation material, although complete removal was not achieved. The results revealed that removal efficacy varied significantly between tooth types, with incisors showing greater obturation material removal, while molars exhibited more pronounced changes in canal volume. These findings provide quantitative data on the retreatability of iodoform‑based materials in primary teeth.

## Data Availability

Data sharing is not applicable to this article as no new data was created. Corresponding authors A.M.-V. ( [amartvac@uax.es](mailto: amartvac@uax.es) ) and M.M.P.-C. ( [mapaz08@ucm.es](mailto: mapaz08@ucm.es) ) can be contacted to request information or the data of the study.

## Abbreviations

CaOH2: Calcium hydroxide
CV: Canal void volume
CBCT: Cone Beam Computed Tomography
µCT: Micro-computed tomography
EDDY: Sonically driven root canal irrigation system
EDTA: Ethylenediaminetetraacetic acid
iVac: Apical negative pressure irrigation and activation system group
NiTi: Nickel–titanium
MPRCF: Mixed Primary Root Canal Filling
MV: Obturation material volume
ΔMV: Obturation material increase
PUI: passive ultrasonic irrigation
RAPID: Reporting stAndards for research in PedIatric Dentistry
RV: Filling removal volume
ΔCV: Root canal void increase
SEM: Scanning electrin microscope
TV: Total volume
VOI: Volume of interest
WL: Working length
ZnO: Zinc oxide
ZOE: Zinc oxide-eugenol cement
